# Myrciaria jaboticaba Bagasse, Peel, and Seed Bioactive-Rich Flours: A Source of Dietary Fibers and Lignocellulosic Biomass for Functional and Technological Food Applications

**DOI:** 10.1021/acs.jafc.4c13114

**Published:** 2025-06-12

**Authors:** Ramon Bocker, Eric Keven Silva

**Affiliations:** 28132 Universidade Estadual de Campinas (UNICAMP), Faculdade de Engenharia de Alimentos (FEA), Rua Monteiro Lobato, 80, Campinas, SP CEP:13083-862, Brazil

**Keywords:** Jabuticaba, pectin, lignin, cellulose, hemicellulose, galacturonic acid

## Abstract

The growing global need for circular bioeconomy processes has driven increasing interest in the valorization of fruit processing byproducts. Jaboticaba (Myrciaria jaboticaba) byproducts, such as bagasse, peel, and seeds, are produced in large quantities by the juice industry and contain valuable technological and bioactive components, including dietary fibers and natural colorants (anthocyanins). While previous studies have primarily focused on their polyphenolic content, limited attention has been given to their dietary fiber and lignocellulosic composition. From this perspective, this study performed a comprehensive physicochemical, structural, and functional characterization of jaboticaba bagasse, peel, and seed flours, with a focus on their soluble and insoluble fibers, as well as their lignocellulosic components, including cellulose, lignin, and hemicellulose. Monosaccharide and disaccharide contents were quantified by HPAEC-PAD. Additionally, Fourier-transform infrared spectroscopy (FTIR), thermogravimetric analysis (TGA), X-ray diffraction (XRD), scanning electron microscopy (SEM), anthocyanin quantification, and total phenolic content analysis were performed to assess the microstructure, technological properties, and bioactive potential. The results revealed high dietary fiber content and structurally diverse lignocellulosic matrices, suggesting potential applications, such as natural thickeners, stabilizers, and functional ingredients in food systems. This study expands the current understanding of jaboticaba byproducts and highlights their relevance for sustainable ingredient development, food innovation, and future applications in functional and clean-label formulations.

## Graphical abstract

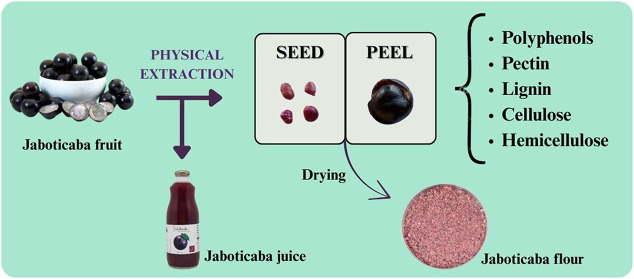

## 1 Introduction

Jaboticaba (Myrciaria cauliflora) is a native fruit of the Brazilian Atlantic rainforest, also found in Paraguay, Argentina, Bolivia, and parts of Central America. This spherical fruit, measuring 2–4 cm in diameter, is characterized by a gelatinous, white pulp and a fragile, thin pericarp that ranges in color from red-black to purple. Each fruit typically contains 1–4 seeds.^1–3^ As a small, dark-colored fruit with antioxidant and other health-promoting properties, jaboticaba is comparable to other fruits, such as grapes, blueberries, blackberries, elderberries, acai berries, and cherries. These fruits share characteristics, such as their rich content of polyphenols, particularly anthocyanins, a compound known for its antioxidant activity. However, its distinctive sensory properties and regional specificity confer jaboticaba a unique profile, offering valuable opportunities for the development of innovative and sustainable products.^3–5^

The primary product derived from jaboticaba is juice, obtained through the maceration of the whole fruit. The production of jaboticaba juice generates substantial quantities of byproducts, primarily peels and seeds.^6^ These materials are often discarded, despite being rich in bioactive compounds (e.g., anthocyanins, tannins, and phenolic acids) and essential nutrients such as dietary fibers.^7–9^ This nutritional and bioactive composition contributes to the antioxidant, antimicrobial, and anti-inflammatory properties of jaboticaba.^10,11^ Additionally, recent *in vivo* studies suggest potential antiproliferative,^12^ protective,^13,14^ antidiabetic,^11^ and antiobesity^15^ effects, primarily attributed to its polyphenolic content. Jaboticaba juice can be consumed fresh or utilized as an ingredient in the development of various products, including liqueurs,^16^ wines,^17,18^ jellies,^19^ nutraceutical beverages,^20^ biopolymer films,^21^ biscuits,^22^ bologna-type sausages,^23^ nonfermented dairy desserts,^24^ and yogurt.^25^ These diverse applications strengthen the jaboticaba value chain and enhance its economic relevance by supporting the development of health-oriented and sensory attractive products and expanding market opportunities.

Figure 1 illustrates the jaboticaba processing chain, and the main products derived from its juice. Although often discarded, jaboticaba bagasse, mainly composed of peel and seeds, are highlighted as coproducts of potential commercial interest due to their rich nutritional and technological profiles.

**Figure 1:**
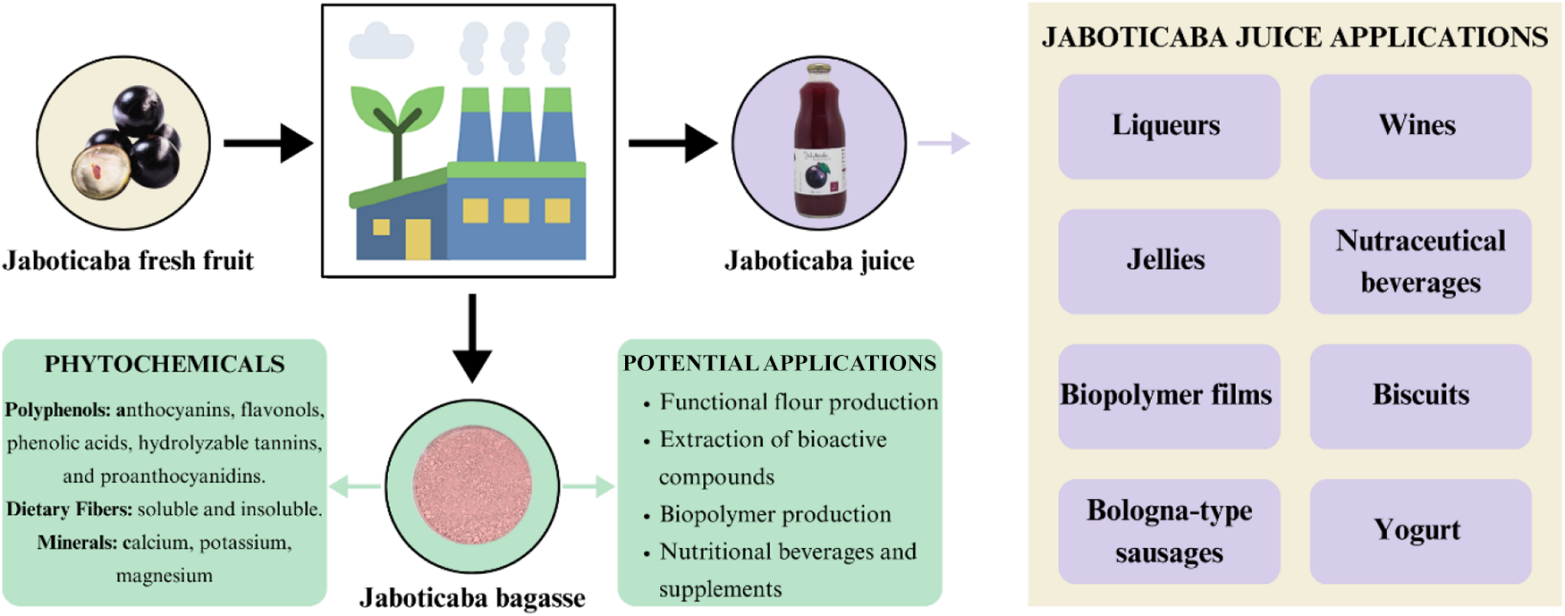
Overview of jaboticaba juice applications and phytochemical potential of jaboticaba bagasse.

Jaboticaba presents several limitations that hinder its commercialization in fresh form. Notably, the fruit exhibits a complicated harvesting process, since it grows directly on the trunk and branches of the tree, alongside high perishability and a prolonged juvenile phase, all of which contribute to logistical and economic challenges. Additionally, the polyphenolic compounds present in jaboticaba, while associated with significant bioactive potential, pose further obstacles for the food industry due to their low thermal, oxidative, and photochemical stability, which limits their direct application in food systems.^6^ Nonetheless, promising avenues for the valorization of agro-industrial residues from jaboticaba processing include functional flours,^26^ bioactive compound extraction,^27^ biopolymer production,^28^ and the development of nutritional beverages and supplements.^29^

The increasing interest in the valorization of agro-industrial waste has led to a growing demand for the characterization of sustainable ingredients, such as jaboticaba byproduct flours.^11,30^ However, current research predominantly focuses on flours derived from peel and seed separately, failing to reflect the industrial reality of jaboticaba residues as a complex mixture of peel, seed, and pulp filtrates.^31^ While significant attention has been given to the bioactive fractions, including flavanols, polyphenols, anthocyanins, and tannins,^27,32,33^ there remains a significant gap in understanding the fiber content and biomass profile of jaboticaba byproducts.

Recent studies have suggested that polysaccharide consumption may be associated with antioxidant,^34^ hypoglycemic,^35^ and antitumor^36^ effects. Additionally, jaboticaba dietary fibers may exhibit prebiotic properties, modulating gut microbiota and stimulating short-chain fatty acid production.^37,38^ Despite this, the cellulose, hemicellulose, and lignin profiles of jaboticaba byproduct flours remain underexplored. Cellulose is a crystalline polymer composed of microfibrils connected through hydrogen bonds and van der Waals forces.^39^ Hemicellulose, on the other hand, is a complex of polysaccharides that includes sugars and pentoses.^40^ Efficient production of fuels and chemicals can be achieved through the utilization of both cellulose and hemicellulose.^41^ Lignin comprises an aromatic polymer characterized by sinapyl and coniferyl alcohol units, with applications that encompass polymer blends, carbon fibers, epoxy resins, polymers, and adhesives.^40,42^ The lignocellulosic profile has significant industrial interest due to the potential valorization of flours within the framework of integrated biorefinery concepts, thereby promoting a circular bioeconomy.^40,43^

An increasingly relevant application of plant-based byproducts is their incorporation into health-oriented food products, serving as natural colorants, thickeners, and stabilizers.^1,11,44^ Within this context, the present study offers a comprehensive physicochemical, structural, and functional characterization of jaboticaba flours, emphasizing their dietary fiber profile and lignocellulosic composition. To the best of our knowledge, this is the first study to simultaneously investigate the flour obtained from jaboticaba bagasse, peel, and seed as distinct and integrated materials. By addressing current knowledge gaps, particularly regarding their polysaccharide and lignin contents, this work contributes to advancing the sustainable use of jaboticaba byproducts as functional ingredients with potential applications across food and nonfood sectors, in alignment with circular bioeconomy and clean-label trends.

## 2 Methodology

### 2.1 Raw Material

Fresh jaboticaba (Myrciaria jaboticaba *(Vell.) O. Berg*) fruits were obtained from a local producer in Campinas, SP, Brazil. The peels, seeds, and pulp were manually separated from part of the fresh fruits. Simultaneously, a jaboticaba-to-water mixture (2:1) was prepared and processed into juice by blending at 50% speed for 3 min in a blender MX1500 (Waring Commercial Inc., United States of America). The mixture was then strained through cheesecloth to collect the bagasse. The bagasse, peel, and seed were individually dried in a convective oven at 40 °C for 48 h. The dried materials were then ground using a blender for 1 min, and the resulting flours—referred to as jaboticaba bagasse flour (JBF), jaboticaba peel flour (JPF), and jaboticaba seed flour (JSF)—were stored at 4 °C.

### 2.2 Proximal Composition

Moisture content was determined by infrared radiation using an infrared balance, model AD-4714A (TECNAL, Brazil), at 105 °C for 15 min. Ash content was determined by incinerating the samples in a muffle furnace at 500–600 °C for 6 h to obtain the fixed mineral residue. Lipid content was measured using the Soxhlet extraction method according to official methods described by AOAC (2023). Protein content was determined using the Dumas method with a nitrogen and protein analyzer, model NDA 701 (Velp Scientifica, Italy). Data analysis was carried out using DUMASoft version 6.1.0. The protein content (%) was calculated by applying a nitrogen-to-protein conversion factor of 6.25.

### 2.3 Particle Size Distribution

The particle size distribution of the samples was determined using a vibratory sieve shaker (Bertel, Brazil) with mesh sizes of 2, 1.41, 0.84, 0.012, and 0.007 mm (Tyler series, United States of America).^45^

### 2.4 Soluble and Insoluble Dietary Fiber

The enzymatic-gravimetric method was used to determine the soluble and insoluble dietary fibers according to the method described by Asp et al.^46^ In this method, the flour was subjected to gelatinization using a heat-stable amylase in a boiling system for 15 min, followed by incubation with pepsin for 1 h at an acidic pH (pH 2.0) and incubation with pancreatin at neutral pH (pH 7.0) for 1 h. The determination of insoluble fibers was carried out by filtration with Celite 545. Soluble fibers were precipitated from the filtrate using 4 volumes of ethanol and recovered on a Celite 545 filter. The percentage of insoluble and soluble fiber was calculated according to eqs 1 and 2.

1 $$Insoluble\;Fiber\;\left(g/100\;g\right)=\frac{\left(D_{1}-I_{1}-B_{1}\right)}{M}\times 100$$

2 $$\mathrm{Soluble}\;\mathrm{Fiber}\;\left(g/100\;g\right)=\frac{\left(D_{2}-I_{2}-B_{2}\right)}{M}\times 100$$

Where: *M* = Sample (g); *D*_1_ = Insoluble residue after drying (g); *I*_1_ = Insoluble residue after incineration (g); *B*_1_ = Blank (without sample) for insoluble fiber (g); *D*_2_ = Soluble residue after drying (g); *I*_2_ = Soluble residue after incineration (g).

### 2.5 Lignocellulosic Biomass Structure

The characterization of lignocellulosic biomass was based on the study reported by Gouveia et al.^47^ To determine the extractives, flour samples were extracted using a Soxhlet apparatus (Behr Labo-Technik, R 106 S, Germany) with a 50:50 v/v mixture of cyclohexane and ethanol for 8 h, followed by water extraction for 48 h. After extraction, the biomass obtained and the extractive-free biomass were dried in an oven at 50 °C for 18 h and at 105 °C for another 18 h, respectively.

The lignin content was assessed through the hydrolysis of the extractive-free samples. Hydrolysis took place in a thermostatic bath (Fisatom 562S, Brazil) at 30 °C for 1 h using sulfuric acid. The samples were then transferred to pressure tubes and autoclaved at 121 °C for 1 h. The hydrolyzed samples were filtered; the insoluble fraction was used for quantifying insoluble lignin, while the filtrate was reserved for soluble lignin analysis. The percentage of insoluble lignin was calculated by subtracting the ash content from the initial mass of flour (after extractives had been removed) and dividing by this initial mass. For soluble lignin analysis, a 5 mL aliquot of the hydrolysis filtrate was mixed with 80 mL of distilled water, and the pH was adjusted to between 12.0 and 12.5 using concentrated NaOH solution. This solution was then transferred to 100 mL volumetric flasks, with volumes adjusted to the mark for UV–vis assays using a UV–visible Spectrophotometer (UV–vis), Brand: Shimadzu, Model: 1800 (Kyoto, Japan) to determine soluble lignin content using eq 3.

3 $$C_{soluble\;lignin}\;\left(\frac{g}{L}\right)=4.187\times 10^{-2}\times \left(A_{T}-A_{pd}\right)-3.279\times 10^{-4}$$

Where: *C*_Soluble lignin_ = concentration of soluble lignin; *A*_T_ = absorbance of the lignin solution with degradation products at 280 nm; Apd = absorbance at 280 nm of sugar decomposition products (furfural and hydroxymethylfurfural).

The methodology for quantifying carbohydrates and organic acids involved high-performance liquid chromatography (HPLC) Dionex Ultimate 3000 (Thermo Scientific, Germany). Detection was performed using a photodiode array detector (DAD-3000), which offered high sensitivity for UV–vis analysis, and a refractive index detector (RI-101, Shodex) that allowed for the detection of analytes based on refractive index. The chromatographic separation was performed using a Bio-Rad Aminex HPX-87H column (7.8 × 300 mm). Chromeleon software (version 6.80, Germany) was employed for system control, data acquisition, and analysis.

Calibration curves were established by injecting solutions of cellobiose, glucose, xylose, arabinose, acetic acid, and formic acid. In the quantification methodology, specific methods were applied based on the concentration range and detector type used for each compound. All compounds detected by the refractive index (RI) detector were quantified within a concentration range of 0.1 to 10 g/L. Sugars quantification were with RI detector a concentration range of 0.001 to 0.1 g/L. Organic acids was utilized with the UV detector set at 210 nm and optimized for concentrations between 0.01 and 0.1 g/L. Additionally, the UV detector set at 278 nm to target these specific compounds at concentrations from 0.01 to 0.1 g/L was applied for the quantification of hydroxymethylfurfural and furfural. These targeted detection and quantification protocols were selected to ensure accurate measurement of each compound within the relevant concentration ranges.

Finally, considering their degradation products, the cellulose and hemicellulose of the samples can be determined from the conversion factors presented in eqs 4 and 5.

4 $$m_{Cellulose}\;\left(g\right)=0.95\times m_{Cellobiose}+1.2\times m_{HMF}+3.09\times m_{Formic\;acid}$$

5 $$m_{Hemicellulose}\;\left(g\right)=0.88\times m_{xylose}+0.72\times m_{acetic\_acid}+0.95\times m_{Cellobiose}+1.2\times m_{furfural}$$

Where: *m*_cellulose_ (g) = mass of cellulose in grams, *m*_cellobiose_ (g) = mass of cellobiose, *m*_hydroxymethylfurfural_ = mass of hydroxymethylfurfural, *m*_formic acid_ (g) = mass of formic acid, *m*_xylose_ = mass of xylose, *m*_acetic acid_ = mass of acetic acid, *m*_furfural_ = mass of furfural.

### 2.6 Monosaccharides and Disaccharides Analysis by High-Performance Anion Exchange Chromatography Coupled to Pulsed Amperometric Detection (HPAEC-PAD)

JBF (0.5 g) and pulp (1.5 g) were extracted with 20 mL of ultrapure water using an Ultra-Turrax homogenizer (IKA T25 digital ULTRA-TURRAX, Germany) at 17,000 rpm for 2 min at room temperature. JBF, JPF, and JSF were hydrolyzed using TFA acid in an autoclave at 120 °C for 30 min, following the methodology described by Pereira et al.^48^ The hydrolysates and aqueous extracts were then centrifuged at 4000*g* for 20 min at 5 °C, and the supernatants were collected. All samples were filtered through 0.22 μm PVDF syringe filters and used for sugar analysis.

The monosaccharides and disaccharides analysis was performed using a HPAEC-PAD system, model DIONEX ICS-5000 (Thermo Fisher Scientific, United State of America), with modifications to the methodology of Pereira et al.^48^ Sugars (rhamnose, arabinose, galactose, glucose, xylose, fructose, galacturonic acid, glucuronic acid, and sucrose) were separated by gradient elution using 0.2 mol/L NaOH (eluent A), ultrapure water (eluent B), and 0.5 mol/L sodium acetate containing 0.2 mol/L NaOH (eluent C) on a CarboPac PA1 column (Thermo Fisher Scientific, United State of America). The gradient program was as follows: 0–22 min, 4% A and 96% B; 22–24 min, 4–50% A and 96–50% B; 24–32 min, 68% B and 32% C; 32–37 min, 100% A; and 37–42 min, 4% A and 96% B.

The column temperature was maintained at 30 °C, with a flow rate of 1.0 mL/min and a sample injection volume of 25 μL. Data was acquired and processed using Chromeleon software version 7.0. Sugars in the samples were identified by comparing retention times with those of authentic standards, and calibration curves constructed with commercial standards were used for quantification.

### 2.7 Morphology and Microstructure

The morphology and surface microstructure of JBF, JSF, and JPF were examined using a benchtop scanning electron microscope, model TM4000Plus (Hitachi, Japan), operating at an acceleration voltage of 5–15 kV. The flour samples were ground to a uniform particle size, placed in the microscope, and high-resolution photomicrographs were captured to analyze their surface morphology.

### 2.8 Fourier Transform Infrared Spectroscopic Analysis

The chemical stability and structural network of the flours were evaluated using a Fourier Transform Infrared (FTIR) spectrometer, model IRPrestige-21, from Shimadzu (Kyoto, Japan). FTIR spectra were recorded over a wavenumber range of 400–4000 cm^–1^. For each sample, a total of 16 scans with Happ-Genzel apodization were averaged, with a resolution of 4 cm^–1^.

### 2.9 X-ray Diffraction

The crystallinity of the flours was determined using an X-ray diffraction (XRD) instrument, model D2 PHASER, from Bruker (Ettlingen, Germany), following the methodology described by Mendes et al.^22^ The analysis was performed at 30 kV and 10 mA, with samples scanned over a 2θ angle range of 5–40°. The scan speed was set to 1°/min, and the results were compared to assess differences in crystalline structure.

### 2.10 Thermogravimetric Analysis (TGA)

The thermal stabilities of the JBF, JPF, and JSF were assessed using a thermogravimetric analyzer (TG-DTA H Shimadzu 60, Shimadzu Corporation, Kyoto, Japan). The analysis was conducted in a nitrogen atmosphere with a flow rate of 10 cm^3^/min, and the samples were heated from 25 to 600 °C at a heating rate of 10 °C/min.

### 2.11 Obtaining Jaboticaba Extract

For phenolic compounds extraction, each flour sample was mixed with an hydroethanolic solution (1:1, v/v) in a plant material-to-solvent ratio of 1:15 (w/w) according to the methodology described by Nunes Mattos et al.^49^ The mixture was continuously stirred at 300 rpm and maintained at 50 °C for 3 h under magnetic stirring in an aluminum foil-covered glass beaker. After extraction, phenolic extracts were separated from the insoluble biomass by centrifugation at 4000 rpm for 10 min. The supernatant was collected and stored at −20 °C for further analysis.

### 2.12 Ion-Trap Profile of Anthocyanins

Based on the approach outlined by Arruda et al.,^50^ chromatographic separation of the sample was performed using a Poroshell 120 SB-Aq column (100 × 2.1 mm i.d., 2.7 μm particle size, Agilent Technologies) at a temperature of 40 °C. The mobile phase was composed of two solvents: A, which contained 0.1% formic acid in water, and B, which was acetonitrile with 0.1% formic acid. A gradient elution method was utilized at a flow rate of 0.45 mL/min, beginning with 5% B for 1 min, followed by a gradual increase to 18% B by 10 min. The gradient then escalated to 70% B by 13 min, and subsequently, a more rapid increase to 100% B was achieved by 15 min. The system was maintained at 100% B for 2 min before reverting to 5% B over the course of 2 min, followed by a 3 min equilibration period at 5% B.

Fragmentation and full scan MS1 of the samples were conducted using an Ion Trap mass spectrometer coupled with UFLC (LC-MSn) from Bruker Daltonics, model amaZon Speed. Data acquisition and qualitative analysis were carried out using DataAnalysis software. The fragmentation patterns of the detected components were compared to established identities reported by Yuzuak et al.^51^

### 2.13 Total Phenolic Content

The total phenolic content (TPC) of the extracts was measured using the Folin-Ciocalteu method, as first described by Singleton and Rossi.^52^ For the assay, 25 μL of diluted sample was combined with 25 μL of Folin-Ciocalteu reagent (1:1 dilution) and 200 μL of 5% (w/v) sodium carbonate solution. The reaction mixture was incubated in the dark at room temperature for 20 min, after which the absorbance was measured at 760 nm using a SpectraMax Mini microplate reader (Molecular Devices, United States of America). A blank control was prepared by replacing the sample with 25 μL of water. A calibration curve was established using gallic acid standards ranging from 5 to 80 μg/mL (*R* ^2^ = 0.993) for TPC quantification.

### 2.14 Total monomeric Anthocyanins

The total monomeric anthocyanin (TMA) content was determined using the differential pH method, as calculated by eqs 6 and 7.^53^ A potassium chloride buffer at pH 1.0 was prepared by mixing 0.3 M solutions of hydrochloric acid and potassium chloride, while a sodium acetate buffer at pH 4.5 was prepared by combining 0.3 M solutions of sodium acetate and acetic acid.

6 $$A={\left({ABS}_{520\;nm}-{ABS}_{700\;nm}\right)}_{pH\;1.0}-{\left({ABS}_{520\;nm}-{ABS}_{700\;nm}\right)}_{pH\;4.5}$$

7 $$C3\mathrm{OG}\;\left(\mathrm{mg}/L\right)=\frac{A\times \mathrm{MW}\times \mathrm{DF}\times 10^{3}}{\varepsilon \times L}$$

Where: MW: Molecular weight of cyanidin-3-glucoside (C3OG) = 449.2 g/mol; DF: Sample dilution factor; ε: Molar extinction coefficient of C3OG = 26900 L/mol/cm; L: Optical path length: 0.87784 cm; 10^3^: Unit conversion (grams to milligrams).

For the analytical procedure, 40 μL of the sample was pipet in triplicate for each buffer pH. To one set of triplicates, 200 μL of the pH 1.0 buffer was added, while the other set received 200 μL of the pH 4.5 buffer. After adding the buffers, the dilutions were allowed to equilibrate in the dark for 15 min. Absorbance readings were then taken using a SpectraMax Mini microplate reader (Molecular Devices, United States of America) at two wavelengths: 520 and 700 nm, with 240 μL of distilled water serving as the blank for both measurements.

### 2.15 Statistical Analysis

Jaboticaba flour samples were produced in duplicate, and both sets were used for all evaluations. Results are presented as mean values with their corresponding standard deviations. Statistical comparisons among the different samples were performed using one-way ANOVA followed by Tukey’s test to determine significant differences between groups, where applicable. Descriptive analyses were performed for qualitative responses obtained from Scanning Electron Microscopy (SEM), FTIR, XRD, and TGA. These evaluations provided a comprehensive discussion of the findings, emphasizing key structural, morphological, and compositional characteristics observed in the samples. Graphical outputs were analyzed to identify trends, correlations, and variations pertinent to the flour samples and their properties.

## 3 Results and Discussion

### 3.1 Proximal Composition

Moisture, lipid, protein, and ash content are key components that influence the functional and technological properties of flours in various food systems.^54^ Table 1 exhibits the values for these components in JBF, JPF, and JSF, revealing significant differences among the flours evaluated. These variations highlight the distinct characteristics of each flour, which can be leveraged for specific applications in the food, pharmaceutical, and cosmetic industries.

**Table 1:** Moisture, Lipid, Protein, and Ash Content of Jaboticaba Bagasse, Peel, and Seed Flours^a^

proximate composition (%)	JBF	JPF	JSF
moisture	9.4 ± 0.1^c^	11.8 ± 0.1^a^	10.5 ± 0.1^b^
lipids	0.91 ± 0.01^a^	0.64 ± 0.01^c^	0.87 ± 0.01^b^
protein	11.3 ± 0.1^a^	9.9 ± 0.1^b^	8.1 ± 0.1^c^
ash	1.83 ± 0.01^c^	2.92 ± 0.04^a^	1.97 ± 0.05^b^

a Results are expressed in mean ± standard deviation. Different letters in the same line indicate significant differences by Tukey’s test at 95% significance (*p*-value <0.05). Results are on a wet basis and represented as % of jaboticaba byproduct flour. JBF: jaboticaba bagasse flour, JPF: jaboticaba peel flour, JSF: jaboticaba seed flour.

JPF exhibited the highest ash content (2.92%), significantly higher (*p*-value <0.001) than both JSF (1.97%) and JBF (1.83%). Higher ash content indicates a greater presence of minerals in the flour. The ash values for JPF were similar to those reported in the literature for flours of Myrciaria jaboticaba pomace, peel, and seed.^7,9,31,55^ The results obtained are also consistent with those reported by Resende et al.,^26^ who evaluated the ash content of 28 jaboticaba peel flours, ranging from 3.34 to 7.87%. The mineral content in the jaboticaba byproducts, particularly in the peel and seed, has been reported in previous studies, including important elements such as potassium (1.006% in the peel, 0.401% in the seed), calcium (0.051% in the peel, 0.017% in the seed), iron (0.0036% in the peel, 0.0013% in the seed), and phosphorus (0.089% in the peel, 0.095% in the seed).^56–58^ These findings support the nutritional relevance of jaboticaba byproducts for potential applications in food and other industries.

The protein content varied significantly among the analyzed fractions, with JBF showing the highest concentration (11.31 ± 0.04%), followed by JPF (9.85 ± 0.07%) and JSF (8.09 ± 0.13%). This variation is due to the composition of each fraction, with JBF retaining more protein material during juice extraction, primarily from the pulp. The protein content in JBF (11.31%) supports its potential use in applications requiring a higher protein load. Ascheri et al.^59^ reported a similar protein level of 11.00% in Myrciaria cauliflora pomace flour, consistent with the results of this study. Additionally, JPF exhibited higher protein levels compared to the 6.13% reported by Almeida et al.^55^ Protein variability across jaboticaba fractions is well-documented, with Resende et al.^26^ reporting a range of 3.81 to 7.27% in 28 samples of jaboticaba peel. Lower protein content, around 2.1%, was reported by Faller et al.^7^ for jaboticaba seed and peel flour, reinforcing the distinct composition of these fractions.

JPF exhibited the highest moisture content (11.75%) compared to JBF (9.4%) and JSF (10.45%). These values are consistent with those reported for jaboticaba powders, such as Myrciaria jaboticaba peel and seed (10.7%, Faller et al.^7^) and Myrciaria jaboticaba peel (11.61%, Nascimento et al.^9^), while being slightly higher than the value found for Myrciaria cauliflora peel (9.46%, Almeida et al.^55^). The moisture content for JBF was lower than that reported by Gurak et al.,^31^ who found a moisture content of 11.5% in Myrciaria cauliflora bagasse powder.

In terms of lipid content, JBF exhibited the highest amount (0.91%), followed by JSF (0.87%) and JPF (0.64%). The lipid content of JPF found in this study was lower than the values reported for Myrciaria cauliflora pomace (0.63%, Gurak et al.^31^), Myrciaria jaboticaba peel and seed (0.7%, Faller et al.^7^), and Myrciaria cauliflora peel (1.22%, Almeida et al.^55^).

### 3.2 Chemical Profile

Figure 2 presents the FTIR spectra of the JBF, JPF, and JSF samples. This analysis provides valuable insights into the chemical structure, molecular interactions, and functional groups of jaboticaba fractions. The bands in the region of 3600–3200 cm^–1^ correspond to O–H stretching, which is attributed to moisture content.^26^ Notably, the strong absorption band observed at 3400 cm^–1^, attributed to O–H stretching vibrations of hydroxyl groups, is influenced by hydrogen bonding within the polysaccharide matrix.^28^ The stretching vibrations of hydroxyl groups around 3450–3400 cm^–1^ also indicate the presence of anthocyanins, phenolic compounds, glycerol, and alcohols in the jaboticaba samples. Additionally, peaks around 2900 cm^–1^ can be attributed to the stretching of carbon–hydrogen bonds within the aromatic rings of phenolic compounds. Similar results were reported by Moura et al.,^60^ who evaluated the FTIR spectra of jaboticaba powders produced through freeze-drying.

**Figure 2:**
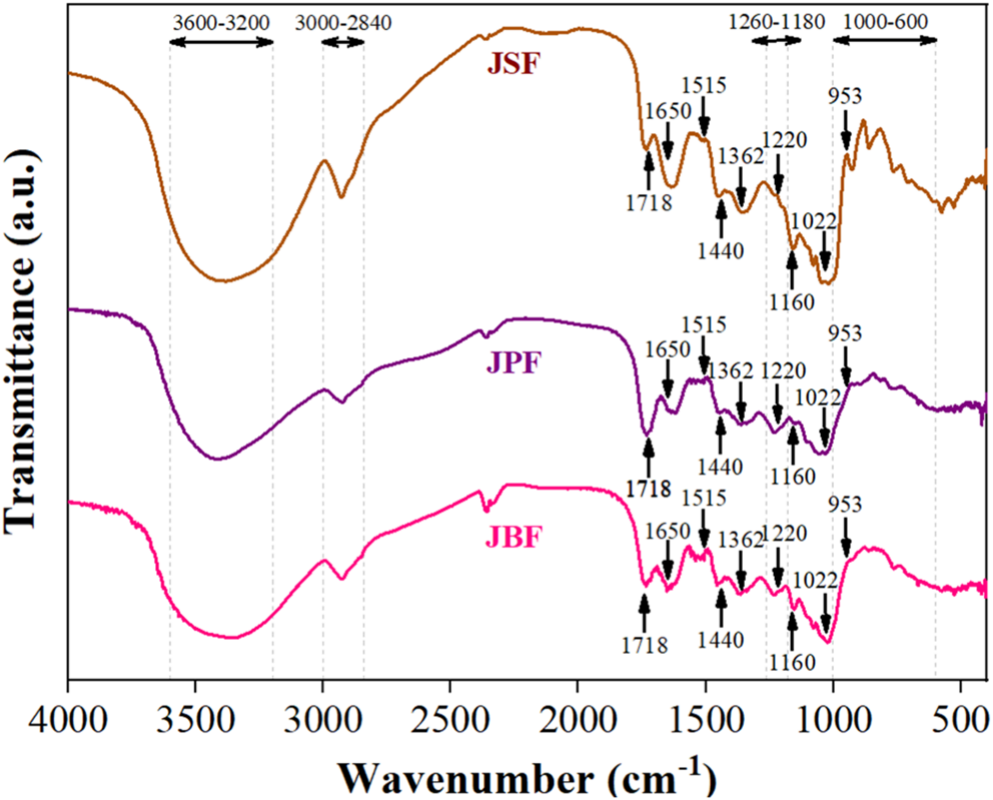
FTIR spectra of jaboticaba fractions (JBF: jaboticaba bagasse flour, JPF: jaboticaba peel flour, JSF: jaboticaba seed flour).

Furthermore, peaks observed at approximately 1722 and 1626 cm^–1^ indicate carbonyl bond deformations, which may be related to the presence of amides, benzopyran aromatic rings, aldehydes, ketones, and carboxylic acids. Specifically, peaks between 1718 and 1725 cm^–1^ could indicate stretching vibrations of the aromatic ring, which are typical for anthocyanins.^27^ Peaks in the range of 1603 to 1613 cm^–1^ correspond to aromatic compounds, such as flavonoids, characterized by phenyl bonds. Peaks near 1200 cm^–1^ suggest C–O angular deformations in phenolic compounds and the pyran ring structures of flavonoids. Peaks between 1100 and 1000 cm^–1^ correspond to the stretching vibrations of glycosides, coupled with ring vibrations and stretching vibrations of lateral groups (C–OH). Finally, peaks within the 1000–600 cm^–1^ region suggest the presence of aromatic rings.^61^

The pectin profile analysis of the jaboticaba fractions indicates that the spectral region of 1760–1650 cm^–1^ is indicative of both esterified and nonesterified carboxyl groups. A recent study by Zhang et al.^28^ investigated jaboticaba peel flour and dietary fibers separately, identifying key pectin-related peaks, including 1440 cm^–1^ (asymmetric stretching modes of methyl esters), as well as 1022 and 953 cm^–1^ (ring vibrations). Within the insoluble dietary fiber spectrum, peaks at 1650 cm^–1^ (N–H angular deformation of amides) and 1760 cm^–1^ (carbonyl bond) are associated with the presence of undigested proteins and enzymes.

Several peaks in the insoluble dietary fiber spectrum are attributed to cellulose, specifically at 1362 cm^–1^ (CH_2_ bending), 1160 cm^–1^ (O–C–O asymmetric stretching), and 1030 cm^–1^ (C–O stretching, C–C stretching). Hemicellulose is represented by peaks at 1362 cm^–1^ (CH_2_ bending, xyloglucan), 1064 cm^–1^ (C–O stretching), and 1041 cm^–1^ (C–O–C stretching, glycosidic). Lignin presents particularly distinctive FTIR values due to the presence of functional groups and structural units typical of the molecule, specifically at 1600, 1515, and 1426 cm^–1^ (aromatic skeleton vibrations); 1215–1220 cm^–1^ (vibrations associated with C–C, C–O, and C=O); and 1370–1375 cm^–1^ (phenolic hydroxyl (OH) groups and aliphatic C–H in methyl groups).^62,63^

### 3.3 Dietary Fibers and Lignocellulosic Biomass

The results presented in Table 2 present the dietary fiber content and lignocellulosic biomass structure among the jaboticaba fractions. The content of dietary fibers and lignocellulosic materials positions JBS, JPF, and JSF as promising technological and functional ingredients. These coproducts, often considered as waste, can be utilized as functional dietary fiber, prebiotic ingredients, stabilizers in food formulations, and network formers in gel systems.

**Table 2:** Soluble and Insoluble Fibers, Lignin, Cellulose, and Hemicellulose of Jaboticaba By-Product Flours^a^

compounds (%)	JBF	JPF	JSF
soluble dietary fibers	6.7 ± 0.1^a^	5.4 ± 0.1^b^	2.4 ± 0.1^c^
insoluble dietary fibers	60.5 ± 1.1^a^	52.8 ± 0.3^c^	56.6 ± 1.2^b^
lignin	21.6 ± 0.1^a^	12.7 ± 0.3^b^	11.6 ± 0.1^c^
cellulose	23.7 ± 0.2^b^	10.0 ± 0.2^c^	49.5 ± 1.2^a^
hemicellulose	10.0 ± 0.2^a^	9.3 ± 0.1^b^	3.1 ± 0.1^c^

a Results are expressed in mean ± standard deviation. Different letters in the same line indicate significant differences by Tukey’s test at 95% significance (*p*-value <0.05). Results are on a dry basis and represented as % of jaboticaba byproduct flour. JPF: jaboticaba peel flour, JBF: jaboticaba bagasse flour, JSF: jaboticaba seed flour.

JBF exhibits the highest content of both soluble dietary fiber (6.67%) and insoluble dietary fiber (60.51%). Notably, a significant increase in both soluble and insoluble fibers in Myrciaria cauliflora pomace has also been reported by Gurak et al.,^31^ who evaluated the fiber content of the pomace, peel, and whole fruit. The ratio of soluble to insoluble fiber in JBF, approximately 90%, exceeds the value reported in study by Gurak et al.,^31^ which indicated a ratio of 80%. This consistent result can be attributed to the concentration of fibrous material retained after the juice filtration process, leading to an increase in both soluble and insoluble fibers in the sample. The fiber content of jaboticaba residues is comparable to that of grape residues, which are considered a rich source of fiber (43–75%).^64^ These findings position JBF as a promising ingredient for enhancing the bulk of food, with potential technological applications as thickeners, stabilizers, gelling agents, and texture modifiers.^65^

JSF exhibits the lowest soluble fiber content (2.42%), while its insoluble fiber content (56.58%) is intermediate compared to the other samples. Although JPF contains less total fiber than JBF, it still provides a significant amount of insoluble dietary fiber (52.83%) and a moderate amount of soluble fiber (5.44%). Previous studies by Faller et al.^7^ reported soluble fiber levels of 1.6% and insoluble fiber levels of 39.3% for a jaboticaba peel and seed mixture flour. Inada et al.^56^ evaluated the peel and seed separately, finding total dietary fiber values of 38.4 and 31.8%, respectively. For soluble dietary fiber, a yield of 6.12% was achieved for jaboticaba byproduct flour, comparable to the value obtained for JBS. Resende et al.^26^ evaluated 28 jaboticaba peel flours, with results ranging from 26.99 to 46.33% for insoluble fibers and 4.41 to 9.27% for soluble fibers. The observed variations can be attributed to differences in cultivation practices, harvesting methods, and fruit processing, all of which influence the chemical profile. Additionally, the duration of exposure to high temperatures during drying and commercial flour production may also affect these results.^66^

Lignin quantification significantly distinguishes JBF (21.6%) from both JPF (12.7%) and JSF (11.6%). This higher lignin content in JBF can be attributed to the concentration of fibrous materials during the juice processing stage. Both sugar cane straw and sugar cane bagasse are recognized as rich lignocellulosic matrices, containing lignin levels ranging from 19 to 32% and 17 to 25%, respectively.^67^ In a comparative study, Watkins et al.^68^ evaluated lignin extraction from various biomasses, reporting values of 34.0% for alfalfa, 22.7% for pine straw, 20.4% for wheat straw, and 14.9% for flax fibers. Thus, JBF represents a sustainable and valuable lignin source. Given the unique structural and functional properties of lignin, this biopolymer is promising for various applications in sustainable materials, including the production of biobased coatings, adhesives, and biofuels.^69–71^

The cellulose content in JSF is remarkably high at 49.5%, compared to 23.7% in JBF and 10% in JPF. This level in JSF surpasses that found in sugar cane straw and sugar cane bagasse, reported as 38–42% and 30–40%, respectively, in a review by Antunes et al.^67^ Additionally, a comparative study by Magalhães et al.^72^ evaluated cellulose in hardwoods (e.g., oak, eucalyptus, acacia, poplar), softwoods (e.g., pine, Douglas fir, spruce), agricultural waste (e.g., barley hull, wheat straw, barley straw, rice straw, rice husks, oat straw, corn stalks, corn cobs, sugar cane bagasse, sorghum straw), and grasses (e.g., switchgrass), with cellulose levels ranging from 35–50%, 40–50%, 25–45%, and 25–40%, respectively. This substantial cellulose concentration in JSF suggests its potential for applications in bioethanol production,^41^ sugar production,^73^ cellulosic pulp, and cellulose nanofibers^74^.

Hemicellulose content also varies across fractions, supporting additional industrial uses; JBF exhibits the highest level at 10%, followed by JPF at 9.3% and JSF at 3.1%. The hemicellulose content of the flours is lower than that presented by sugar cane straw and sugar cane bagasse, which have values of 19–35% and 19–28%, respectively.^67^ Among the matrices evaluated by Yashika and Chopra^75^—including wheat straw, rice straw, corn cob, nut shells, hardwood stem, cotton seed hair, softwood stem, bamboo, corn stover, barley straw, switch grass, miscanthus, and poplar—hemicellulose levels range from 20–38%. The exception is Cotton Seed Hair, with a lower range of 5–20%, which includes the values for JPF and JSF.

### 3.4 Structural Carbohydrate Profile

Acid hydrolysis was performed to evaluate the structural sugars, present as complex polymers, and the quantification of JBF, JPF, and JSF was carried out using HPAEC-PAD. Table 3 presents the sugars profiling, including rhamnose, arabinose, galactose, glucose, xylose, fructose, galacturonic acid, glucuronic acid, and total sugars.

**Table 3:** Monosaccharide and Disaccharide Content of Jaboticaba Bagasse, Peel, and Seed Flours^a^

components	molecular weight (g/mol)	molecular formula	ret. time (min)	JBF (%)	JPF (%)	JSF (%)
rhamnose	164.16	C_6_H_12_O_5_	8.92	1.65 ± 0.03^a^	0.74 ± 0.02^b^	n.d.
arabinose	150.13	C_5_H_10_O_5_	9.62	9.62 ± 0.29^a^	6.55 ± 0.22^b^	1.30 ± 0.03^c^
galactose	180.16	C_6_H_12_O_6_	12.12	8.34 ± 0.29^a^	5.76 ± 0.24^b^	1.79 ± 0.01^c^
glucose	180.16	C_6_H_12_O_6_	13.43	37.51 ± 1.49^a^	10.04 ± 0.40^b^	36.24 ± 1.69^a^
xylose	150.13	C_5_H_10_O_5_	15.3	4.33 ± 0.12^a^	2.32 ± 0.03^b^	n.d.
galacturonic acid	194.14	C_6_H_10_O_7_	29.87	12.20 ± 0.61^a^	7.91 ± 0.23^b^	1.42 ± 0.03^c^
glucuronic acid	194.14	C_6_H_10_O_7_	31.05	0.96 ± 0.01^a^	0.35 ± 0.01^c^	0.75 ± 0.01^b^
total sugars				74.81 ± 2.78^a^	33.66 ± 1.14^c^	41.49 ± 1.75^b^

a Results are expressed in mean ± standard deviation. Different letters in the same line indicate significant differences by Tukey’s test at 95% significance (*p*-value <0.05). Results are on a dry basis and represented as % of jaboticaba byproduct flour. n.d. = not detected; JPF: jaboticaba peel flour; JBF: jaboticaba bagasse flour; JSF: jaboticaba seed flour.

The analysis of JBF reveals a complex sugar profile, reflecting the mix of peel, pulp, and seeds in this residue. Glucose, the predominant carbohydrate at 37.51%, highlights the potential of JBF as a valuable fiber source for industrial applications, such as bioethanol production and structural materials. Arabinose, at 9.62%, is notably high, suggesting significant amounts of arabinogalactans and pectin, particularly from the peel. Galactose, at 8.35%, reflects the presence of galacturans and pectin. Galacturonic acid, averaging 12.20%, indicates a high concentration of homogalacturonan pectin, important for gel formation and thickening properties.^76^ This sugar profile underscores the potential for industrial applications requiring high viscosity and gel-forming capacity of JBF sample.^77^ The lower glucuronic acid levels (0.96%) suggest a reduced proportion of hemicelluloses compared to other fruit parts. The galacturonic acid to glucuronic acid ratio (12.77:1) confirms the predominance of homogalacturonan pectin, reinforcing the potential for gelling and thickening applications of JBF sample.^78^

In the JSF sample, glucose was identified as the predominant monosaccharide, with an average concentration of 36.24%. This elevated glucose content reinforces the discussion about the substantial concentration of cellulose in JSF, highlighting its role as a major energy source and structural component. Galactose and arabinose were present in lower amounts, averaging 1.79 and 1.30%, respectively. These sugars indicate the presence of pectin and polysaccharides that reinforce the cellular structure of the seed. The low levels of glucuronic acid (0.75%) and galacturonic acid (1.42%) suggest minimal presence of uronates typically found in hemicelluloses and pectin.^76,79^ The ratio of galacturonic acid to glucuronic acid (1.91:1) indicates a predominance of homogalacturonan pectin in the seed.^78^

In the JPF sample, glucose was present at an average concentration of 1.34%, significantly lower than JSF. However, arabinose was found in significant quantities, with an average level of 6.55%. This high concentration suggests a notable presence of arabinogalactans and pectin, which are critical for the structural and functional properties of the peel. The high arabinose content implies potential applications for the peel in products requiring gelling or thickening agents. Galactose was also present in considerable amounts, ranging from 5.76%, indicating the presence of galactans and pectin that contribute to the structure and consistency of the peel. The significant presence of galacturonic acid, with average levels ranging from 7.91%, suggests that the peel is rich in pectin, consistent with the soluble fiber determination.^80^ The lower concentration of glucuronic acid (0.35%) indicates a smaller number of hemicelluloses associated with pectin compared to the seed and bagasse. The ratio of galacturonic acid to glucuronic acid (approximately 22.69:1) indicates a predominance of homogalacturonan pectin in the peel.^76,78^

Quantitative analysis of free and soluble sugars in JBF and jaboticaba pulp was conducted using HPAEC-PAD. Given that JBF is derived from juice processing and contains pulp fractions, its sugar profile is relevant for potential industrial applications. In the pulp sample, the primary sugars identified included fructose (6.70%), glucose (5.13%), and sucrose (0.71%), with glucose and fructose being the predominant sugars. The total sugar content in pulp was 12.54%, indicating that the pulp is a rich source of soluble sugars, consistent with the carbohydrate-rich profile typically found in fruit pulps. As expected, JBF displayed a lower total sugar concentration of 5.95%. Fructose was the most abundant sugar in JBF, averaging 3.49%, followed by glucose at 2.46%. The reduced sugar content in the bagasse compared to the pulp can be attributed to its fibrous nature and the presence of peel and seed fractions in the sample.

### 3.5 X-ray Diffraction

Figure 3 presents the XRD pattern analysis of JBF, JPF, and JSF. Each peak along the horizontal axis corresponds to a crystalline plane within the samples, representing the reflection of X-rays by specific atomic arrangements within the crystalline structure. XRD characterization typically differentiates between crystalline and amorphous structures, identified by sharp/narrow peaks and broad/dispersed peaks, respectively. For compound identification, the positions of the peaks (2θ) are compared against reference X-ray diffraction data. JSF exhibited sharper and more intense peaks, indicative of a more ordered crystalline structure. The prominent peak between 17 and 18° 2θ in the XRD diffractogram of jaboticaba seed flour suggests a significant amount of crystalline cellulose and potentially starch. Bendit^81^ reported that a peak around 20° 2θ is characteristic of β-sheet structures, which Manzoor et al.^82^ in two varieties of apple seed flour, highlighting the predominance of crystalline regions related to β-sheet formation. The broad peak observed in the 20–25° 2θ range is typically associated with the amorphous regions of semicrystalline polysaccharides and dietary fibers, such as amorphous cellulose and hemicellulose.

**Figure 3:**
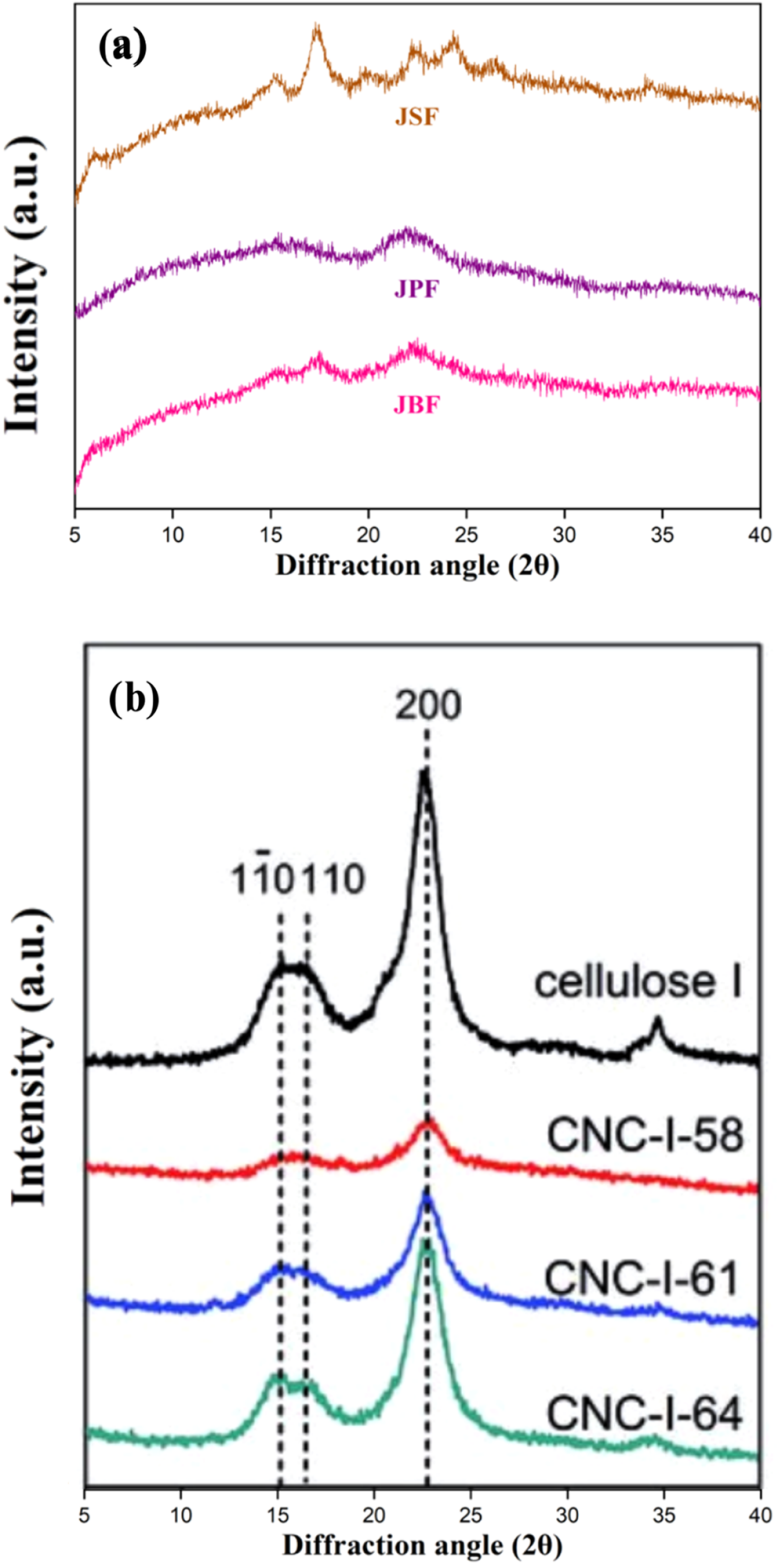
(a) X-ray diffraction (XRD) patterns of the jaboticaba byproduct samples. (b) XRD patterns for cellulose I and cellulose nanocrystals (CNCs) from literature.^83^ JBF: jaboticaba bagasse flour, JPF: jaboticaba peel flour, JSF: jaboticaba seed flour. CNC-I-58, CNC-I-61, and CNC-I-64 correspond to nanocrystals obtained from cellulose I by sulfuric acid hydrolysis at concentrations of 58, 61, and 64 wt %, respectively.

### 3.6 Thermal Stability

Thermal stability is a key factor for the technological applications of jaboticaba byproduct flour ingredients particularly because it is rich in bioactive compounds such as phenolics, flavonoids, and dietary fibers. Structural changes caused by heat exposure can lead to degradation of bioactive, loss of antioxidant activity, and alterations in the physicochemical properties of the flours, compromising its functionality as a high-value ingredient.

Figure 4 presented the TGA for each flour evaluated. Results show significant differences in the samples weight loss profiles. The TGA peaks indicate the maximum rate of weight loss at each stage, highlighting distinct decomposition events for each sample.

**Figure 4:**
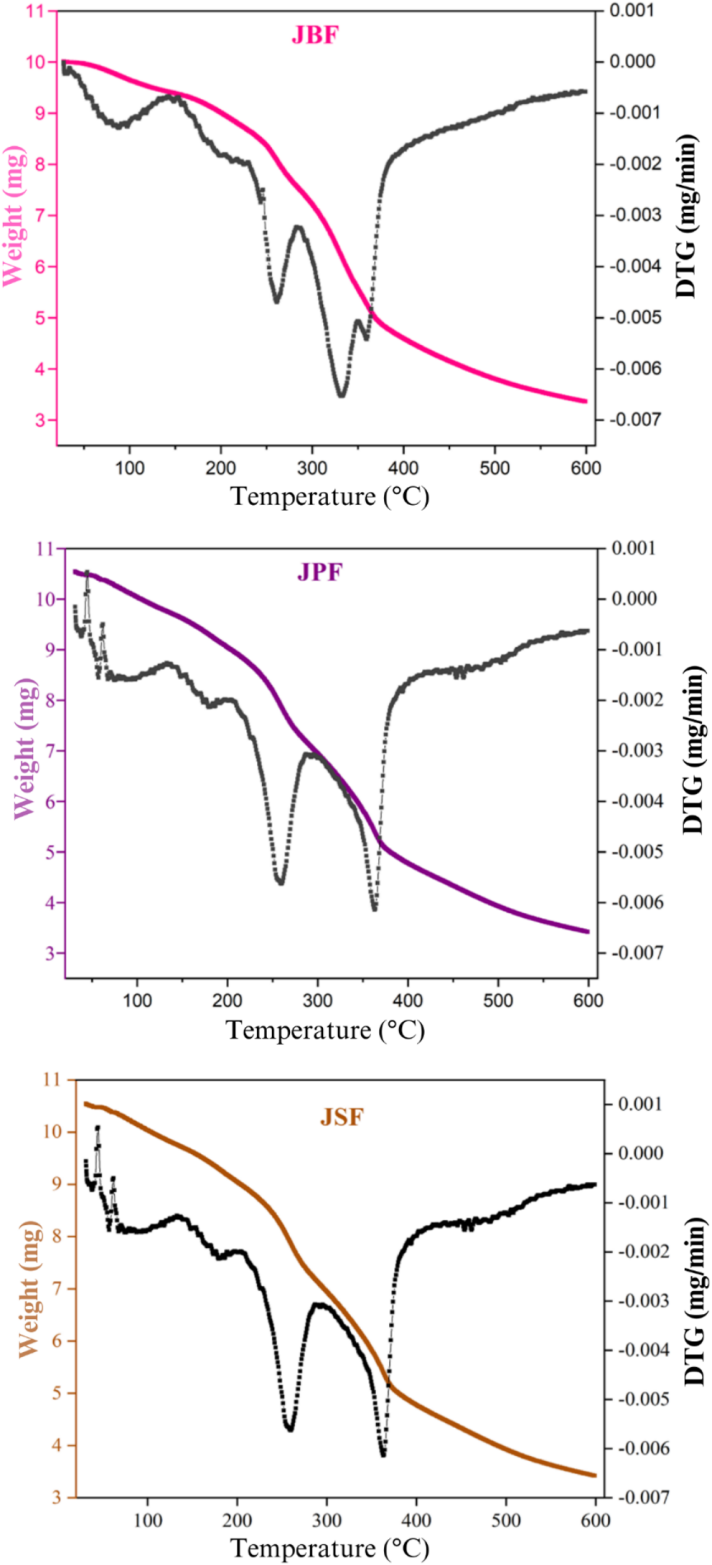
Thermogravimetric analysis (TGA) of the jaboticaba byproduct samples. JBF: jaboticaba bagasse flour, JPF: jaboticaba peel flour, JSF: jaboticaba seed flour.

For practical applications of the flours, the water retention capacity within the working range of 50–150 °C is of particular importance. The initial stability phase (I), characterized by negligible weight loss, demonstrated distinct trends among the samples. The onset and offset temperatures during this phase varied (JBF: 34–114 °C; JPF: 36–111 °C; JSF: 27–126 °C), leading to different mass losses (JBF: 6.09%; JPF: 7.22%; JSF: 9.27%). These results provide critical insights into the thermal behavior of the flours under conditions commonly encountered in industrial processes. The lower mass loss of JBF within this range suggests its potential for applications requiring minimal moisture evaporation under moderate thermal treatments, such as precooked foods or formulations that maintain stability during the drying stage.^84,85^

The second phase of thermal degradation (II), where significant mass loss begins, also showed variation between the samples. The temperature range and percentage of weight loss during the initial decomposition phase were different for each sample (JBF: 150–272 °C, 18.9%; JPF: 135–185 °C, 7.1%; JSF: 165–256 °C, 11.8%). A higher onset temperature and wider temperature range in this phase indicate greater thermal stability and mass retention. In this regard, JPF shows limitations compared to the other samples, with the lowest initial temperature and narrowest decomposition range, initiating accelerated decomposition earlier. Conversely, JBF exhibits a more gradual mass loss, implying a more controlled thermal degradation profile as the system absorbs thermal energy. The superior thermal stability of JBF, indicated by its wider temperature range in the second degradation phase, highlights its suitability for intermediate thermal processes, such as baking or extrusion.^22,84,86^

The third phase, characterized by accelerated decomposition (III), marks a rapid weight loss as thermal degradation proceeds at full scale. The temperature ranges and weight loss percentages for this phase also differed across the samples (JBF: 287–342 °C, 19.2%; JPF: 205–271 °C, 18.7%; JSF: 273–352 °C, 37.1%). Notably, JSF displayed the highest mass loss during accelerated decomposition, suggesting lower thermal stability compared to the other samples.

Finally, the residual phase (IV) represents the conclusion of the thermal degradation process, where mass loss stabilizes. The plateau observed in this phase varied for each sample (JBF: 350–395 °C, 22.0%; JPF: 296–396 °C, 33.9%; JSF: 400–495 °C, 12.1%), reflecting differences in the residual inorganic content or other nonvolatile compounds. The significantly higher residual mass of JPF suggests a greater proportion of thermally stable compounds, whereas JSF, with a lower residual mass, indicates a more extensive breakdown of its constituents. The rapid decomposition observed in JSF may limit its application under severe thermal conditions but can be advantageous for systems requiring texturization or controlled release of volatile compounds.^84^

### 3.7 Anthocyanins Identification, Total Monomeric Anthocyanins, and Total Phenolic Compounds

The ion trap analysis of jaboticaba fractions revealed distinct anthocyanin profiles across the JPF, JBF, and JSF samples. Cyanidin-3-*O*-glucoside (retention time (r.t.) = 6.7 min) was tentatively identified in all samples (JBF, JPF, and JSF), with *m*/*z* = 449 and MS/MS fragments at 287 and 288. This anthocyanin is the most abundant in jaboticaba, as previously reported in recent studies.^1,12,33,49,56^

Delphinidin-3-*O*-glucoside (r.t. = 5.6 min), with *m*/*z* = 465 and MS/MS fragments at 303 and 304, was tentatively identified in JBF, JPF, and JSF. This anthocyanin is the second most abundant in jaboticaba. Pelargonidin-*O*-hexoside (r.t. = 7.3 min), with *m*/*z* = 433 and MS/MS fragments at 271 and 272, was detected in JBF and JPF but was absent in JSF. This is consistent with findings from Tarone et al.^33^ Quatrin et al.,^87^ who identified it in jaboticaba peel. Additionally, petunidin (r.t. = 6.8 min), with *m*/*z* = 479 and MS/MS fragments at 317 and 318, was detected, aligning with reports from Romualdo et al.^14^ Peonidin (*m*/*z* = 465, MS/MS = 303 and 304), as reported by Tarone et al.,^33^ was absent in JSF. Cyanidin-3-(6″-coumaroyl) glucoside (r.t. = 7.4 min), with *m*/*z* = 595 and MS/MS = 287, was identified in JBF and JPF but not detected in JSF.

To gain deeper insight into the implications of the distinct anthocyanin profiles observed among the jaboticaba fractions, the total phenolic content (TPC) and total monomeric anthocyanins (TMA) were quantified in JBF, JPF, and JSF. The results are presented in Table 4.

**Table 4:** Total Phenolic Content (TPC) and Total Monomeric Anthocyanins (TMA) in Jaboticaba By-Product Flours (JBF, JPF, and JSF)^a^

bioactive compound	JBF	JPF	JSF
TMA (mg C3OG/100 g)	155 ± 7^b^	253 ± 29^a^	19 ± 1^c^
TPC (mg GAE/100 g)	3031 ± 545^b^	6924 ± 300^a^	3389 ± 267^b^

a Results are expressed in mean ± standard deviation. Different letters in the same line indicate significant differences by Tukey’s test at 95% significance (*p*-value <0.05). Results are on a dry basis and represented as % of jaboticaba by-product flour. JPF: jaboticaba peel flour, JBF: jaboticaba bagasse flour, JSF: jaboticaba seed flour. GAE: gallic acid equivalent. C3OG: Cyanidin-3-*O*-glucoside.

Cyanidin-3-*O*-glucoside (C3OG), a predominant anthocyanin, showed considerable variation across the fractions. JBF exhibited 155 ± 7 mg C3OG/100 g of flour, while JPF had a higher concentration of 253 ± 29 mg C3OG/100 g, consistent with the value of 258 mg C3OG/100 g reported by study by Nunes Mattos et al.^49^ under similar extraction conditions. In contrast, JSF contained only 19 ± 1 mg C3OG/100 g of flour, emphasizing the significantly higher anthocyanin concentration in the peel relative to other fractions. The lower anthocyanin content in JBF compared to JPF may be attributed to the reduced anthocyanin levels in the pulp fraction. Complementary analysis of the pulp’s anthocyanin content showed no detectable amounts by spectrophotometric methods.

The total phenolic content in the samples demonstrated notable variation, with JPF showing the highest concentration at 6924 ± 300 mg GAE/100 g, which significantly surpasses the values found in JBF and JSF. Both JBF and JSF presented lower amounts of phenolic compounds, measuring 3031 ± 545 mg GAE/100 g and 3389 ± 267 mg GAE/100 g, respectively, with no statistically significant difference (*p*-value = 0.399) between the two. These findings suggest that the phenolic profile of JPF is richer in bioactive compounds compared to JSF and JBF. Furthermore, the phenolic content observed in all samples is notably higher than the range reported by Bueno et al.,^88^ which varied from 1785 to 5141 mg GAE/100 g. The higher phenolic content in this study underscores the potential of jaboticaba byproducts, particularly peel flour, as a source of antioxidant compounds, which are valuable for developing functional foods with health-promoting properties.

Previous studies have reported considerable tannin concentrations in different jaboticaba fractions, with values reaching 4.8% in the peel, 1.16% in the pulp, and 6.5% in the seed.^89^ Tannins in jaboticaba are known to bind to proteins and digestive enzymes, reducing nutrient availability and digestibility. However, they also exhibit antioxidant and potential therapeutic properties, including anti-inflammatory, anticancer, and antidiabetic effects, indicating a dual role in both limiting nutrient absorption and promoting health.

Although the elevated phenolic content in JPF is promising, it is important to consider that the bioavailability of these compounds may be limited due to their possible binding to dietary fibers or formation of insoluble complexes, which can reduce their physiological efficacy. Therefore, studies assessing the bioaccessibility and bioavailability of these compounds are essential to better understand their actual health benefits.

### 3.8 Particle Size Distribution, Morphological Characterization, and Implications for Functional Applications and Sustainable Utilization

The jaboticaba byproduct flours (JBF, JPF, and JSF) were also characterized by their particle size distributions. JPF contained the highest proportion of coarse particles (>2 mm), while JSF showed the highest content of fine particles (<0.0117 mm). Intermediate sieve sizes revealed varying distributions, with JPF consistently displaying more coarse material and JSF a predominance of finer fractions. JBF exhibited an intermediate profile, reflecting its mixed composition. These findings highlight the distinct granulometric characteristics of each flour, which may influence their behavior in technological applications.

Scanning electron microscopy (SEM) images (Figure 5) further support these observations, revealing structural differences among the flours. JPF exhibited a laminar and fibrous morphology, characteristic of peel-derived material. JSF presented a more compact and granular structure, consistent with its seed origin. JBF, which consists of a combination of seed and peel, displayed particle structures where seed and peel components could be distinguished. At higher magnification (1000×), a denser outer layer was evident in JBF, likely associated with retained pulp fibers, indicating lower surface porosity compared to JSF.

**Figure 5:**
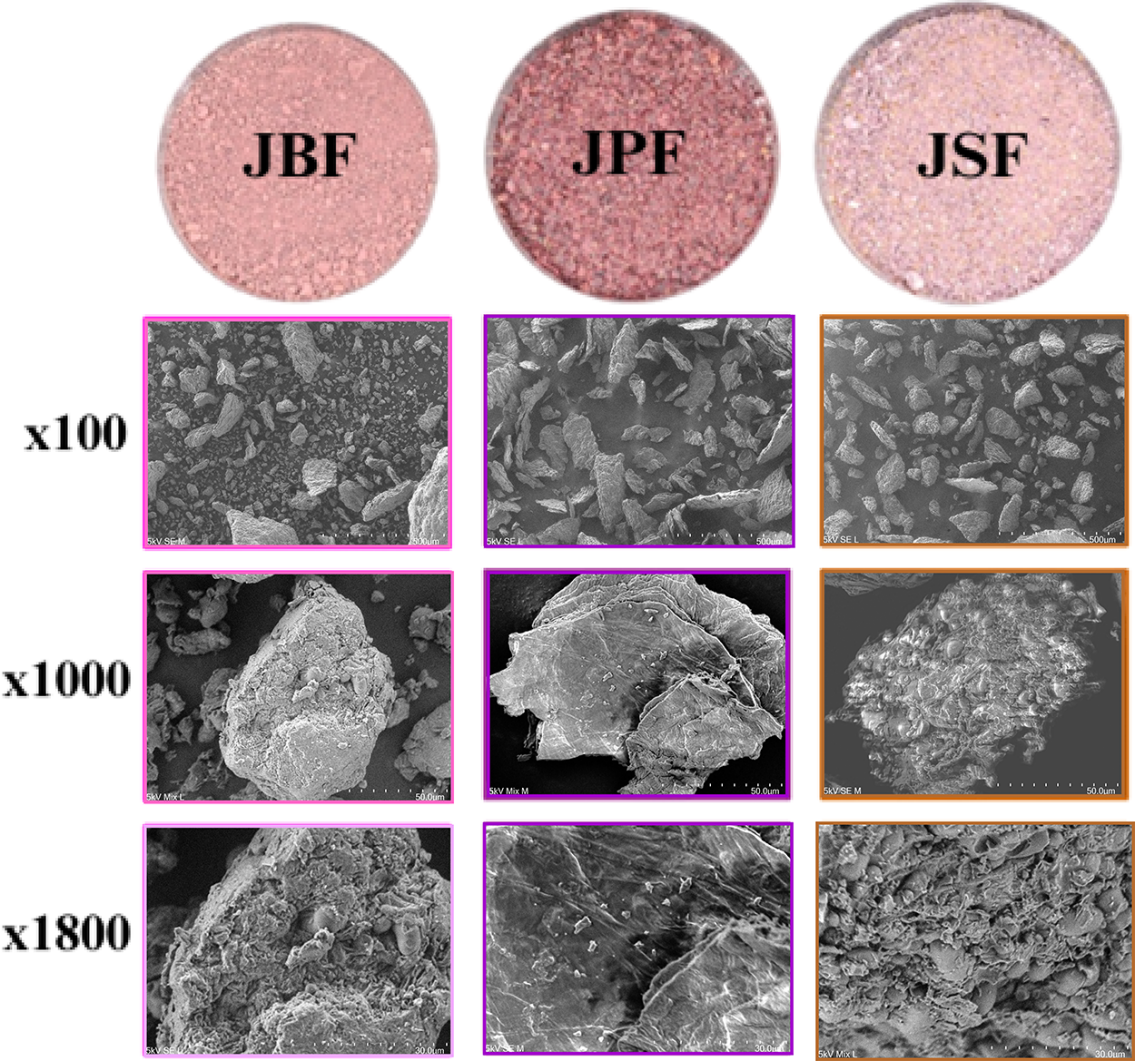
High-resolution scanning electron microscopy (SEM) image revealing the intricate surface morphology of the samples.

These differences in particle size and composition may influence the flours’ functionality in food systems. Coarser particles, as observed in JPF, can enhance water-holding capacity and slow down hydration rates, contributing to texture modulation in food products. This is particularly advantageous in bakery and extrusion processes, where gradual moisture absorption is often desired. Moreover, the high content of anthocyanins and soluble fibers in the peel highlights the potential of JPF as a natural thickener and colorant in food applications. In contrast, the finer particles predominant in JSF provide a larger surface area, which may improve dispersion in aqueous systems, enhance emulsion and gel stability, and facilitate the release of encapsulated bioactive compounds. Therefore, particle size can be considered a tunable parameter to adjust texture, hydration behavior, and potentially the bioavailability of functional compounds in food matrices.^6^

Overall, the comprehensive characterization of jaboticaba byproduct flours underscores their potential as versatile ingredients rich in anthocyanins, dietary fiber, and lignocellulosic biomass. While direct application testing was not conducted, the observed structural and compositional properties suggest that each fraction may offer specific functional advantages depending on the target formulation. Furthermore, the full utilization of jaboticaba processing residues contributes to reducing agro-industrial waste and supports sustainable strategies for the development of value-added products.

## References

[1] Albuquerque B. R., Pereira C., Calhelha R. C., José Alves M., Abreu R. M. V., Barros L., Oliveira M. B. P. P., Ferreira I. C. F. R. (2020). Jabuticaba residues (Myrciaria jaboticaba (Vell.) Berg) are rich sources of valuable compounds with bioactive
properties. Food Chem..

[2] Bocker R., Silva E. K. (2024). Anthocyanin-rich
jaboticaba fruit: Natural source of
bioactive and coloring ingredients for nutraceutical food applications. Trends Food Sci. Technol..

[3] da
Silva Monteiro Wanderley B. R., de Lima N. D., Deolindo C. T. P., Kempka A. P., Moroni L. S., Gomes V. V., Gonzaga L. V., Costa A. C. O., de Mello Castanho Amboni R. D., de Sena
Aquino A. C. M., Fritzen-Freire C. B. (2024). Impact of pre-fermentative maceration
techniques on the chemical characteristics, phenolic composition,
in vitro bioaccessibility, and biological activities of alcoholic
and acetic fermented products from jaboticaba (Plinia trunciflora). Food Res. Int..

[4] Wu S.-B., Dastmalchi K., Long C., Kennelly E. J. (2012). Metabolite
Profiling
of Jaboticaba (Myrciaria cauliflora) and Other Dark-Colored Fruit Juices. J. Agric.
Food Chem..

[5] Chua L. S., Chan Y. L., Tay Z. Y., Soo J. (2023). Water-soluble propolis
extract as a natural preservative for jaboticaba juice. Food Biosci..

[6] Benvenutti L., Moura F. M., Zanghelini G., Barrera C., Seguí L., Zielinski A. A. (2025). An Upcycling
Approach from Fruit Processing By-Products:
Flour for Use in Food Products. Foods.

[7] Faller A. L. K., Duarte P. A., Paes J. d. M., Kamp F., Fialho E., Monteiro M. (2023). Jabuticaba (Myrciaria jaboticaba) peel and seed powder associated
with bioprocessing improves functional
and nutritional quality of whole-wheat bread. Int. J. Food Sci. Technol..

[8] Miranda B. M., Di-Medeiros M. C. B., Batista K. A., Carbonero E. R., Fernandes K. F., Silva F. A. (2020). A galactose-rich heteropolysaccharide
extracted from “jaboticaba” (Plinia cauliflora) peels. Carbohydr. Polym..

[9] Nascimento R. d. P. d., Rizzato J. S., Polezi G., Moya A. M. T. M., Silva M. F., Machado A. P. d. F., Franchi Junior G. C., Borguini R. G., Santiago M. C. P. d.
A., Paiotti A. P. R., Pereira J. A., Martinez C. A. R., Marostica Junior M. R. (2023). Freeze-dried
jaboticaba (Myrciaria jaboticaba) peel
powder, a rich source of anthocyanins and phenolic acids, mitigates
inflammation-driven colorectal cancer in mice. Food Biosci..

[10] Filho A. V., Avila L. B., Lacorte D. H., Martiny T. R., Rosseto V., Moraes C. C., Dotto G. L., Carreno N. L. V., da
Rosa G. S. (2022). Brazilian Agroindustrial Wastes as a Potential Resource
of Bioative Compounds and Their Antimicrobial and Antioxidant Activities. Molecules.

[11] Benvenutti L., Zielinski A. A. F., Ferreira S. R. S. (2021). Jaboticaba (Myrtaceae cauliflora)
fruit and its by-products: Alternative sources for new foods and functional
components. Trends Food Sci. Technol..

[12] Paludo M. C., de Oliveira S. B. P., de Oliveira L. F., Colombo R. C., Gómez-Alonso S., Hermosín-Gutiérrez I., Prata R., Lima A. F., Filho J. T., Ballus C. A., Godoy H. T. (2022). Phenolic composition
of peels from different Jaboticaba species determined by HPLC-DAD-ESI/MSn
and antiproliferative activity in tumor cell lines. Curr. Plant Biol..

[13] Nogueira-Lima E., Lamas C. d. A., Baseggio A. M., do Vale J. S. F., Maróstica
Junior M. R., Cagnon V. H. A. (2020). High-fat diet effects on the prostatic
adenocarcinoma model and jaboticaba peel extract intake: protective
response in metabolic disorders and liver histopathology. Nutr. Cancer.

[14] Romualdo G. R., de Souza I. P., de Souza L. V., Prata G. B., Fraga-Silva T. F. d.
C., Sartori A., Borguini R. G., Santiago M. C. P. d.
A., Fernandes A. A. H., Cogliati B., Barbisan L. F. (2021). Beneficial effects
of anthocyanin-rich peels of Myrtaceae fruits on chemically-induced
liver fibrosis and carcinogenesis in mice. Food
Res. Int..

[15] Brito T. G. d. S., Silva A. P. S. A. d., Cunha R. X. d., Fonseca C. S. M. d., Araújo T. F. d. S., Campos J. K. d. L., Nascimento W. M., Araújo H. D. A.
d., Silva J. P. R. e., Tavares J. F., Santos B. S. d., Lima V. L. d. M. (2021). Anti-inflammatory,
hypoglycemic, hypolipidemic, and analgesic activities of Plinia cauliflora (Mart.) Kausel (Brazilian grape)
epicarp. J. Ethnopharmacol..

[16] de
Andrade Neves N., Gómez-Alonso S., García-Romero E., Hermosín-Gutiérez I., Ferreira da Silva I., Stringheta P. C. (2022). Chemical composition of jabuticaba (Plinia jaboticaba)
liquors produced from cachaça and cereal alcohol. LWT.

[17] Lima R., Silva M. V. T., Gomes B. A., Macedo E. H. B. C., Santana M. N., Amaral A. C. F., Silva J. R. A., Corrêa P. G., Godoy R. L. O., Santiago M. C. P. A., Leitão S. G., Simas R. C., Carneiro C. S., Rodrigues I. A. (2023). Chemical
Profile and Hematoprotective Activity of Artisanal Jabuticaba (*Plinia jabuticaba*) Wine and Derived Extracts. Fermentation.

[18] Macedo E. H. B. C., Santos G. C., Santana M. N., Jesus E. F. O., de
Araújo U. B., Anjos M. J., Pinheiro A. S., Carneiro C. S., Rodrigues I. A. (2021). Unveiling the physicochemical properties and chemical
profile of artisanal jabuticaba wines by bromatological and NMR-based
metabolomics approaches. LWT.

[19] Romão P. V. M., Palozi R. A. C., Guarnier L. P., Silva A. O., Lorençone B. R., Nocchi S. R., Moura C., Lourenço E. L. B., Silva D. B., Gasparotto
Junior A. (2019). Cardioprotective effects of Plinia cauliflora (Mart.) Kausel in a rabbit model
of doxorubicin-induced heart failure. J. Ethnopharmacol..

[20] da
Silva J. Y. P., do Nascimento H. M. A., de Albuquerque T. M. R., Sampaio K. B., Dos Santos Lima M., Monteiro M., Leite I. B., da Silva E. F., do Nascimento Y. M., da Silva M. S., Tavares J. F., de Brito Alves J. L., de Oliveira M. E. G., de Souza E. L. (2024). Revealing the Potential
Impacts of Nutraceuticals Formulated with Freeze-Dried Jabuticaba
Peel and Limosilactobacillus fermentum Strains Candidates for Probiotic
Use on Human Intestinal Microbiota. Probiotics
Antimicrob. Proteins.

[21] Ribeiro
Sanches M. A., Camelo-Silva C., Tussolini L., Tussolini M., Zambiazi R. C., Becker Pertuzatti P. (2021). Development,
characterization and optimization of biopolymers films based on starch
and flour from jabuticaba (Myrciaria cauliflora) peel. Food Chem..

[22] Mendes D. d. C. S., Asquieri E. R., Batista R. D., de Morais C. C., Ramirez Ascheri D. P., de Macêdo I. Y.
L., de Souza
Gil E. (2021). Microencapsulation of jabuticaba extracts (Myrciaria
cauliflora): Evaluation of their bioactive and thermal
properties in cassava starch biscuits. LWT.

[23] dos
Santos B. A., da Fontoura A. M., Correa L. P., Pinton M. B., Padilha M., Fracari P. R., Ribeiro S. R., Wagner R., Cichoski A. J., Barin J. S., Campagnol P. C. B. (2023). Jabuticaba
peel extract and nisin: A promising combination for reducing sodium
nitrite in Bologna-type sausages. Meat Sci..

[24] de
Sousa M. C., dos Santos W. M., Orleans da Silva J. M., Ramos F. P., de Freitas A. S., Almeida Neta M. C., dos Santos K. M. O., Alonso Buriti F. C., Florentino E. R. (2021). Non-fermented
Dairy Desserts with Potentially Probiotic Autochthonous Lactobacilli
and Products from Peel of Jabuticaba (Myrciaria cauliflora). Probiotics Antimicrob. Proteins.

[25] Machado K. R., Tulini F. L., Guimarães J. D., Moraes I. C., Ditchfield C., Lima C. G., Silva V. L., Favaro-Trindade C. S. (2023). Production
and Evaluation of Yogurt Colored with Anthocyanin-Rich Pigment Prepared
from Jabuticaba (Myrciaria cauliflora Mart.) Skin. Processes.

[26] Resende L. M., Oliveira L. S., Franca A. S. (2020). Characterization
of jabuticaba (Plinia cauliflora) peel
flours and prediction of
compounds by FTIR analysis. LWT.

[27] Santos S. S. D., ParaÍSo C. M., Costa S. C. D. A., Ogawa C. Y. L., Sato F., Madrona G. S. (2022). Recovery
of bioactive
compounds from an agro-industrial waste: extraction, microencapsulation,
and characterization of jaboticaba­(Myrciaria cauliflora Berg) pomace as a source of antioxidant. An.
Acad. Bras. Ciências.

[28] Zhang S., Zhang Q., Wang T., Li C., Tang L., Xiao L. (2024). Response Surface Optimization of
Polysaccharides from Jaboticaba
(Myrciaria cauliflora [Mart.] O.Berg)
Fruits: Ultrasound-Assisted Extraction, Structure Properties, and
Antioxidant/Hypoglycemic Activities. Chem. Biodiversity.

[29] Rigolon T. C. B., Borges L. L. R., Nascimento A. L. A. A., Fernandes J. G., Marins J. C. B., Martins E., Campelo P. H., Stringheta P. C. (2024). Study of
the stability of hydroelectrolytic sports beverages enriched with
phenolic extract from jaboticaba peel or blueberry pulp. Food Humanity.

[30] do
Nascimento R. S., Pedrosa L. d. F., Diethelm L. T. H., Souza T., Shiga T. M., Fabi J. P. (2020). The purification of pectin from commercial
fruit flours results in a jaboticaba fraction that inhibits galectin-3
and colon cancer cell growth. Food Res. Int..

[31] Gurak P. D., De Bona G. S., Tessaro I. C., Marczak L. D. F. (2014). Jaboticaba Pomace
Powder Obtained as a Co-product of Juice Extraction: A Comparative
Study of Powder Obtained from Peel and Whole Fruit. Food Res. Int..

[32] Plaza M., Batista Â. G., Cazarin C. B. B., Sandahl M., Turner C., Östman E., Maróstica Júnior M. R. (2016). Characterization
of antioxidant polyphenols from Myrciaria jaboticaba peel and their effects on glucose metabolism and antioxidant status:
A pilot clinical study. Food Chem..

[33] Tarone A. G., Goupy P., Ginies C., Marostica M. R., Dufour C. (2021). Advanced characterization of polyphenols
from Myrciaria jaboticaba peel and
lipid protection in
in vitro gastrointestinal digestion. Food Chem..

[34] Resende L. M., Franca A. S. (2023). Jabuticaba (Plinia
sp.) Peel as a Source of Pectin:
Characterization and Effect of Different Extraction Methods. Foods.

[35] Xie C., Gao W., Li X., Luo S., Chye F. Y. (2022). Study on the hypolipidemic
properties of garlic polysaccharide in vitro and in normal mice as
well as its dyslipidemia amelioration in type2 diabetes mice. Food Biosci..

[36] Ye G., Li J., Zhang J., Liu H., Ye Q., Wang Z. (2021). Structural
characterization and antitumor activity of a polysaccharide from Dendrobium
wardianum. Carbohydr. Polym..

[37] Alezandro M. R., Granato D., Genovese M. I. (2013). Jaboticaba
(Myrciaria
jaboticaba (Vell.) Berg), a Brazilian grape-like fruit,
improves plasma lipid profile in streptozotocin-mediated oxidative
stress in diabetic rats. Food Res. Int..

[38] Lacerda
Massa N. M., Dantas Duarte Menezes F. N., de Albuquerque T. M. R., de Oliveira S. P. A., Lima M. d. S., Magnani M., de Souza E. L. (2020). Effects of digested jabuticaba (Myrciaria
jaboticaba (Vell.) Berg) by-product on growth and
metabolism of Lactobacillus and Bifidobacterium indicate prebiotic
properties. LWT.

[39] Han Z., Zhu H., Cheng J.-H. (2022). Structure
modification and property improvement of
plant cellulose: Based on emerging and sustainable nonthermal processing
technologies. Food Res. Int..

[40] Rajesh
Banu J., Preethi, Kavitha S., Tyagi V. K., Gunasekaran M., Karthikeyan O. P., Kumar G. (2021). Lignocellulosic biomass based biorefinery:
A successful platform towards circular bioeconomy. Fuel.

[41] Broda M., Yelle D. J., Serwańska K. (2022). Bioethanol Production from Lignocellulosic
Biomass-Challenges and Solutions. Molecules.

[42] Gaspar R., Fardim P. (2023). Lignin-based materials for emerging advanced applications. Curr. Opin. Green Sustainable Chem..

[43] Faria G. M. L., Silva E. K. (2024). Pulsed electric
field, ultrasound and microwave heating
based extraction techniques for valorization of pomegranate peel by-products:
A review. J. Environ. Chem. Eng..

[44] Bortolini D. G., Maciel G. M., Fernandes I. d. A. A., Rossetto R., Brugnari T., Ribeiro V. R., Haminiuk C. W. I. (2022). Biological
potential and technological
applications of red fruits: An overview. Food
Chem. Adv..

[45] Zabot G. L., Moraes M. N., Meireles M. A. A. (2014). Influence of the bed geometry on
the kinetics of rosemary compounds extraction with supercritical CO2. J. Supercrit. Fluids.

[46] Asp N. G., Johansson C. G., Hallmer H., Siljestroem M. (1983). Rapid enzymic
assay of insoluble and soluble dietary fiber. J. Agric. Food Chem..

[47] Gouveia E. R., Nascimento R. T. d., Souto-Maior A. M., Rocha G. J. d. M. (2009). Validation
of methodology for the chemical characterization of sugarcane bagasse. Chem. New.

[48] Pereira G. A., Silva E. K., Peixoto
Araujo N. M., Arruda H. S., Meireles M. A. A., Pastore G. M. (2019). Obtaining
a novel mucilage from mutamba seeds exploring
different high-intensity ultrasound process conditions. Ultrason. Sonochem..

[49] Nunes
Mattos G., Pessanha de Araújo Santiago M. C., Sampaio Doria Chaves A. C., Rosenthal A., Valeriano Tonon R., Correa Cabral L. M. (2022). Anthocyanin Extraction from Jaboticaba
Skin (Myrciaria cauliflora Berg.) Using
Conventional and Non-Conventional Methods. Foods.

[50] Arruda H. S., Silva E. K., Pereira G. A., Angolini C. F. F., Eberlin M. N., Meireles M. A. A., Pastore G. M. (2019). Effects of high-intensity ultrasound
process parameters on the phenolic compounds recovery from araticum
peel. Ultrason. Sonochem..

[51] Yuzuak S., Ballington J., Li G., Xie D.-Y. (2024). High-Performance
Liquid Chromatography–Quadrupole Time-of-Flight Tandem Mass
Spectrometry-Based Profiling Reveals Anthocyanin Profile Alterations
in Berries of Hybrid Muscadine Variety FLH 13–11 in Two Continuous
Cropping Seasons. Agronomy.

[52] Singleton V. L., Rossi J. A. (1965). Colorimetry of Total
Phenolics with Phosphomolybdic-Phosphotungstic
Acid Reagents. Am. J. Enol. Vitic..

[53] Lee J., Durst R. W., Wrolstad R. E., Collaborators (2005). Determination
of Total Monomeric
Anthocyanin Pigment Content of Fruit Juices, Beverages, Natural Colorants,
and Wines by the pH Differential Method: Collaborative Study. J. AOAC Int..

[54] Eckhardt S., Franke H., Schwarz S., Lachenmeier D. W. (2022). Risk Assessment
of Coffee Cherry (Cascara) Fruit Products for Flour Replacement and
Other Alternative Food Uses. Molecules.

[55] Almeida R. L. J., dos Santos Pereira T., Almeida R. D., Santiago Â. M., de Lima Marsiglia W. I. M., Nabeshima E. H., de Sousa Conrado L., de Gusmão R. P. (2021). Rheological
and technological characterization
of red rice modified starch and jaboticaba peel powder mixtures. Sci. Rep..

[56] Inada K. O. P., Oliveira A. A., Revorêdo T. B., Martins A. B. N., Lacerda E. C. Q., Freire A. S., Braz B. F., Santelli R. E., Torres A. G., Perrone D., Monteiro M. C. (2015). Screening of the chemical composition
and occurring antioxidants in jabuticaba (Myrciaria
jaboticaba) and jussara (Euterpe edulis) fruits and
their fractions. J. Funct. Foods.

[57] Alezandro M. R., Dubé P., Desjardins Y., Lajolo F. M., Genovese M. I. (2013). Comparative
study of chemical and phenolic compositions of two species of jaboticaba: Myrciaria jaboticaba (Vell.) Berg and Myrciaria cauliflora (Mart.) O. Berg. Food Res. Int..

[58] Lima A. d. J. B., Corrêa A. D., Dantas-Barros A. M., Nelson D. L., Amorim A. C. L. (2011). Sugars, organic
acids, minerals and
lipids in jabuticaba. Rev. Bras. Fruticult..

[59] Ascheri D. P. R., Ascheri J. L. R., Carvalho C. W. P. d. (2006). Characterization
of jaboticaba bagasse meal and functional properties of extruded products. Food Sci. Technol..

[60] Moura M. d. S., da Silva Gomes da Costa B., Giaconia M. A., de Andrade R. R., Braga A. R. C., Braga M. B. (2023). Jaboticaba
powders production by
freeze-drying: Influence of octenyl succinic anhydride-modified starch
concentrations over anthocyanins and physical properties. J. Food Process Eng..

[61] Krysa M., Szymańska-Chargot M., Zdunek A. (2022). FT-IR and FT-Raman
fingerprints of flavonoids – A review. Food Chem..

[62] Boeriu C. G., Bravo D., Gosselink R. J. A., van Dam J. E. G. (2004). Characterisation
of structure-dependent functional properties of lignin with infrared
spectroscopy. Ind. Crops Prod..

[63] Raspolli
Galletti A. M., D’Alessio A., Licursi D., Antonetti C., Valentini G., Galia A., Nassi o Di Nasso N. (2015). Midinfrared
FT-IR as a Tool for Monitoring Herbaceous Biomass Composition and
Its Conversion to Furfural. J. Spectrosc..

[64] Spinei M., Oroian M. (2021). The Potential of Grape
Pomace Varieties as a Dietary
Source of Pectic Substances. Foods.

[65] Carlsen H., Pajari A. M. (2023). Dietary fiber -
a scoping review for Nordic Nutrition
Recommendations 2023. Food Nutr Res..

[66] Basanta M. F., Ponce N. M. A., Rojas A. M., Stortz C. A. (2012). Effect of extraction
time and temperature on the characteristics of loosely bound pectins
from Japanese plum. Carbohydr. Polym..

[67] Antunes F., Mota I. F., da Silva Burgal J., Pintado M., Costa P. S. (2022). A review
on the valorization of lignin from sugarcane by-products: From extraction
to application. Biomass Bioenergy.

[68] Watkins D., Nuruddin M., Hosur M., Tcherbi-Narteh A., Jeelani S. (2015). Extraction and characterization of
lignin from different
biomass resources. J. Mater. Res. Technol..

[69] Kaur R., Thakur N. S., Chandna S., Bhaumik J. (2021). Sustainable Lignin-Based
Coatings Doped with Titanium Dioxide Nanocomposites Exhibit Synergistic
Microbicidal and UV-Blocking Performance toward Personal Protective
Equipment. ACS Sustainable Chem. Eng..

[70] Li R. J., Gutierrez J., Chung Y.-L., Frank C. W., Billington S. L., Sattely E. S. (2018). A lignin-epoxy resin derived from biomass as an alternative
to formaldehyde-based wood adhesives. Green
Chem..

[71] Singh R., Shukla A., Tiwari S., Srivastava M. (2014). A review on
delignification of lignocellulosic biomass for enhancement of ethanol
production potential. Renewable Sustainable
Energy Rev..

[72] Magalhães S., Fernandes C., Pedrosa J. F. S., Alves L., Medronho B., Ferreira P. J. T., Rasteiro M. D. (2023). Eco-Friendly Methods for Extraction
and Modification of Cellulose: An Overview. Polymers.

[73] Zhu J. Y., Pan X. (2022). Efficient
sugar production from plant biomass: Current status, challenges,
and future directions. Renewable Sustainable
Energy Rev..

[74] Freville E., Pescheux-Sergienko J., Mujica R., Rey C., Bras J. (2024). Novel technologies
for producing tridimensional cellulosic materials for packaging: A
review. Carbohydr. Polym..

[75] Yashika, L. ; Chopra, M. , Review on Hemicellulose: Abundance, Extraction and Application. ymerdigital 2024, 23 (07).

[76] Wang Q., Wang Z., Song J., Xu K., Tian W., Cai X., Mo J., Cao Y., Xiao J. (2023). Homogalacturonan enriched
pectin based hydrogel enhances 6-gingerol’s colitis alleviation
effect via NF-κB/NLRP3 axis. Int. J. Biol.
Macromol..

[77] Du Y., Zhang S., Waterhouse G. I. N., Zhou T., Xu F., Wang R., Sun-Waterhouse D., Wu P. (2024). High-intensity pulsed
electric field-assisted acidic extraction of pectin from citrus peel:
Physicochemical characteristics and emulsifying properties. Food Hydrocolloids.

[78] Freitas C. M. P., Coimbra J. S., Souza V. G., Sousa R. C. (2021). Structure
and Applications
of Pectin in Food, Biomedical, and Pharmaceutical Industry: A Review. Coatings.

[79] Assifaoui A., Hayrapetyan G., Gallery C., Agoda-Tandjawa G. (2024). Exploring
techno-functional properties, synergies, and challenges of pectins:
A review. Carbohydr. Polym. Technol. Appl..

[80] Beukema M., Faas M. M., de Vos P. (2020). The effects
of different dietary
fiber pectin structures on the gastrointestinal immune barrier: impact
via gut microbiota and direct effects on immune cells. Exp. Mol. Med..

[81] Bendit E. G. (1960). A Quantitative
X-Ray Diffraction Study of the Alpha-Beta Transformation in Wool Keratin. Text. Res. J..

[82] Manzoor M., Singh J., Gani A. (2021). Characterization
of apple (Malus
domestica) seed flour for its structural and nutraceutical potential. LWT.

[83] Gong J., Li J., Xu J., Xiang Z., Mo L. (2017). Research on cellulose
nanocrystals produced from cellulose sources with various polymorphs. RSC Adv..

[84] Gharsallah A., Layachi A., Louaer A., Satha H. (2021). Thermal degradation
kinetics of Opuntia Ficus Indica flour and talc-filled poly (lactic
acid) hybrid biocomposites by TGA analysis. J. Compos. Mater..

[85] Silva E. K., Azevedo V. M., Cunha R. L., Hubinger M. D., Meireles M. A. A. (2016). Ultrasound-assisted
encapsulation of annatto seed oil: Whey protein isolate versus modified
starch. Food Hydrocolloids.

[86] de
Lima Paula P., Lemos A. S. d. O., Queiroz L. S., Rocha V. N., Coimbra E. S., Fabri R. L., Denadai Â. M. L. (2023). Supramolecular
complexes between Plinia cauliflora (DC.) Kausel extracts and β-cyclodextrin: Physicochemical
characterization and antioxidant and anti-inflammatory properties. J. Drug Delivery Sci. Technol..

[87] Quatrin A., Pauletto R., Maurer L. H., Minuzzi N., Nichelle S. M., Carvalho J. F. C., Maróstica M. R., Rodrigues E., Bochi V. C., Emanuelli T. (2019). Characterization
and quantification
of tannins, flavonols, anthocyanins and matrix-bound polyphenols from
jaboticaba fruit peel: A comparison between Myrciaria trunciflora
and M. jaboticaba. J. Food Compos. Anal..

[88] Bueno T. M., Queiroz F., Santos J. C. C. D., Furtado M. L. B., Schiassi M. C. E. V., Borges S. V., Figueiredo J. A. (2024). Sequential extraction of anthocyanins
and pectin from jabuticaba (Plinia cauliflora) peel: Peel pretreatment effect and ultrasound-assisted extraction. An. Acad. Bras. Ciências.

[89] Abe L. T., Lajolo F. M., Genovese M. I. (2012). Potential
dietary sources of ellagic
acid and other antioxidants among fruits consumed in Brazil: Jabuticaba
(Myrciaria jaboticaba (Vell.) Berg). J. Sci. Food Agric..

